# Swimming Mode of Two Interacting Squirmers under Gravity in a Narrow Vertical Channel

**DOI:** 10.3390/e24111564

**Published:** 2022-10-30

**Authors:** Geng Guan, Jianzhong Lin, Deming Nie

**Affiliations:** 1 State Key Laboratory of Fluid Power Transmission and Control, Zhejiang University, Hangzhou 310027, China; 2 Zhejiang Provincial Engineering Research Center for the Safety of Pressure Vessel and Pipeline, Ningbo University, Ningbo 315211, China; 3 Institute of Fluid Mechanics, China Jiliang University, Hangzhou 310018, China

**Keywords:** interacting squirmers, lattice Boltzmann method, swimming modes, vertical channel

## Abstract

The swimming mode of two interacting squirmers under gravity in a narrow vertical channel is simulated numerically using the lattice Boltzmann method (LBM) in the range of self-propelling strength 0.1 ≤ *α* ≤ 1.1 and swimming type −5 ≤ *β* ≤ 5. The results showed that there exist five typical swimming patterns for individual squirmers, i.e., steady upward rising (SUR), oscillation across the channel (OAC), oscillation near the wall (ONW), steady upward rising with small-amplitude oscillation (SURO), and vertical motion along the sidewall (VMS). The parametric space (*α*, *β*) illustrated the interactions on each pattern. In particular, the range of oscillation angle for ONW is from 19.8° to 32.4° as *α* varies from 0.3 to 0.7. Moreover, the swimming modes of two interacting squirmers combine the two squirmers’ independent swimming patterns. On the other hand, the pullers (*β* < 0) attract with each other at the initial stage, resulting in a low-pressure region between them and making the two pullers gradually move closer and finally make contact, while the result for the pushers (*β* > 0) is the opposite. After the squirmers’ interaction, the squirmer orientation and pressure distribution determine subsequent squirmer swimming patterns. Two pushers separate quickly, while there will be a more extended interaction period before the two pullers are entirely separated.

## 1. Introduction

In recent years, researchers have attracted considerable interest from self-propelled microorganisms because of their wide range of applications in nature and industrial processes. Microorganisms in nature swim under their energy in different forms [1,2,3,4], and the swimming Reynolds number (*Re*) of microorganisms is usually minimal because the scale of microorganisms is tiny [5]. The squirmer has been studied as a typical driving model of microorganisms and self-propelled particles [6,7]. Based on their driving model, the squirmer could be classified into the pusher, puller, and neutral squirmer [8]. The pusher and puller have different swimming characteristics, e.g., the swimming speed of the pusher increased significantly with increasing Re, but the swimming speed of the puller was not related to Re [9].

The behavior of squirmers swimming near walls is also of great interest. The swimming pattern of squirmers depended on the combined action of self-propulsion, gravity, and hydrodynamic interactions with walls [10]. Several distinct swimming behaviors exist, such as touching the wall and then moving away from the wall, swimming along the wall with periodic oscillations, and swimming parallel to the wall [11]. Fadda et al. [12] studied the dynamics of a chiral swimmer sedimenting on a flat plate. They revealed that different states of gravitational intensity produced different dynamics, but only the wall perpendicular to the direction of gravity affected the swimmer’s dynamics. Recently, Ouyang and Lin [13] investigated the behavior of a settling micro-swimmer in a narrow vertical channel and found four typical locomotive patterns, i.e., the vertical motion, attracted oscillatory, oscillatory, and upward motion. The patterns were related to *Re* and the density ratio of a swimmer to fluid. Moreover, the swimming pattern near the boundary of axially symmetric squirmers in an inertia-less Newtonian fluid with a no-slip interface was theoretically proposed by Ishimoto and Gaffney [14], indicating that the tangential squirmer is a relatively simple framework that enables predictions and classifications of the complexities associated with axially symmetric squirmers swimming near the boundary.

The study of microbial interactions provides a better understanding of microbial motility behavior. Several investigations had empirically indicated that the hydrodynamic interactions between microorganisms played a vital role in defining their dynamics, such as the dancing alga *Volvox* [15], changes in direction between two swimming [16], dynamic clustering in suspensions of motile bacteria [17], and a new form of collective swimming bacteria dynamics [18]. Saintillan and Shelley [19] studied the orientational order and dynamics numerically in suspensions of self-locomoting slender rods, considering the far-field interactions. They found that nematic suspensions of swimming particles were unstable at long wavelengths. Lushi et al. [20] used a spiral vortex state in confined suspensions of *Bacillis subtilis* to study the dynamics of swimmer suspensions. For near-field hydrodynamics between two interacting swimming microorganisms, Papavassiliou and Alexander [21] provided exact solutions of the Stokes equations for squirmers near a no-slip surface, which allowed the hydrodynamic interactions of swimming microscopic organisms to be determined in the near-field. Li et al. [11] numerically investigated the hydrodynamic interactions of swimming organisms at small to intermediate *Re*. They found that the inertial effects changed the contact time and dispersion dynamics of a pair of pusher swimmers and triggered the hydrodynamic attraction of two pullers. Their findings had significant potential implications for studying predator–prey interactions. Therefore, it is essential to understand the effect of inertia on the hydrodynamic interaction between two swimmers. Recently, More and Ardekani [22] studied numerically the interactions between a pair of model swimming organisms in configurations of approaching each other and moving side by side with finite inertia in a linearly density-stratified fluid and observed four types of interactions. Qi et al. [23] studied the hydrodynamic properties of a squirmer type of self-propelled particle in a simple shear flow. They found that increasing the swimming intensity of the squirmer would induce the squirmer to make a periodic and stable motion at a specific distance from the wall. However, no reports on the interaction between the two rising squirmers have been published. Not only do microorganisms have common sedimentation motions, but rising motions are also indispensable because of their physical characteristics [24] and survival needs [25].

Despite the significant progress made in the past, the study of swimming microorganisms in the presence of external forces is still insufficient, especially under the influence of gravity. A suspension of photosynthetic algae, denser than water, may have average swimming upward in still water. Based on this phenomenon, Hilla et al. [26] found that when the density of the upper regions of the suspension becomes more significant than the lower region it may lead to biological connections. Pedley [27] also reported that the upward swimming behavior of algae is influenced by gravity. In other words, the bottoms of algae are heavy, so they are affected by gravitational torque when they are titled. In addition, gravity can also determine the swimming behavior of some microorganisms, which are usually immersed in water and can sense gravity. For example, the weighted leverage within *Loxodes* can control the swimming direction [28], and *Euglena gracilis* (a photosynthetic flagellate) uses gravity to adjust its vertical position in the water to avoid excessive solar radiation [29]. Studying how microorganisms respond to external gravitational fields and how they interact under gravity can provide qualitative assistance in designing artificially intelligent swimming robots. With this in mind, our objective is to comprehensively analyze the motion behavior and interactions of two rising squirmers under the influence of gravity. The swimming patterns of two interacting squirmers under gravity in a narrow vertical channel are simulated numerically using the LBM for different self-propelling strengths and swimming types to understand the collective swimming behavior of microorganisms better. Unlike before, our work focuses on two light squirmers. We hope our results will provide new insights into the dynamical characteristics of squirmers.

### 1.1. Lattice Boltzmann Model

In this work, we use the single relaxation time LBM [30,31,32] to deal with the particle motion in the flow because of its streamlined programming and sufficient accuracy. The discrete lattice Boltzmann equation is expressed as follows:

(1) $$f_{i}(x+e_{i}\Delta t,t+\Delta t)-f_{i}(x,t)=-\frac{1}{\tau }[f_{i}(x,t)-f_{i}{}^{\left(0\right)}(x,t)],$$

where *f_i_*(***x***, *t*) is the distribution function for the microscopic velocity; ***e***_i_ is in the *i*th direction; *f_i_*^(0)^(***x***, *t*) is the equilibrium distribution function; Δ*t* is the time step of the simulation, and *τ* is the relaxation time related to the fluid viscosity *ν*.

The density *ρ* and velocity ***u*** of the fluid are computed using the following formulations:

(2) $$\rho =\sum_{i}f_{i},\rho u=\sum_{i}f_{i}e_{i},$$

The equilibrium distribution function, *f_i_*^(0)^(***x***, *t*), is defined as follows:

(3) $$f_{i}{}^{\left(0\right)}(x,t)=\omega_{i}\rho [1+\frac{3e_{i}\cdot u}{c^{2}}+\frac{9{\left(e_{i}\cdot u\right)}^{2}}{2c^{4}}-\frac{3u^{2}}{2c^{2}}].$$

where *c* = Δ*x*/Δ*t* and Δ*x* is the lattice spacing; for simplicity, Δ*x* and Δ*t* are both fixed at 1, which is common in using LBM. In addition, the weights are *ω*_0_ = 4/9, *ω*_1–4_ = 1/9, and *ω*_5–8_ = 1/36.

For two-dimensional (2-D) simulations, the D2Q9 lattice model with nine velocities proposed by Qian et al. [30] is used. Note that the speed of sound has the relation *c_s_* = *c*/3^1/2^, and the discrete velocity vectors are given:

(4) $$e_{i}=\left\{ \begin{array}{l} (0,\;0)\;\;\;\;\;\;\;\;\;\;\;\;\;\;\;\;\;\;\;\;\;\;\;\;\mathrm{for}\;i=0\;\\ (\pm 1,\;0)c,(0,\;\pm 1)c\;\mathrm{for}\;i=1\;\mathrm{to}\;4\;\\ (\pm 1,\;\pm 1)c\;\;\;\;\;\;\;\;\;\;\;\;\;\;\mathrm{for}\;i=5\;\mathrm{to}\;8 \end{array}\right.\;.$$

By performing a Chapman–Enskog expansion, the macroscopic mass and momentum equations in the low Mach number limit can be recovered,

(5) $$\frac{\partial \rho }{\partial t}+\nabla \cdot (\rho u)=0,$$

(6) $$\frac{\partial (\rho u)}{\partial t}+\nabla \cdot (\rho uu)=-\nabla p+\rho \nu \nabla^{2}u,$$

The kinematic viscosity of the fluid is determined using the equation *ν* = *c_s_*^2^(*τ* − 0.5)Δ*t*.

### 1.2. Squirmer Model

**Figure 1:**
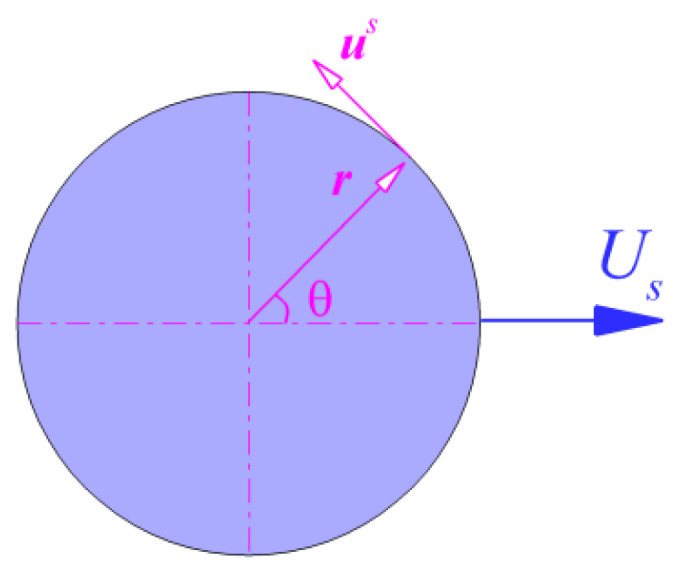
Schematic diagram of the unit vectors.

To enable the self-propelling behavior of particles, we used the squirmer model, which Blake [7] obtained and modified based on the results from Lighthill [6] to simulate the motion of self-propelled particles. According to Blake [7], the slip velocity at one point on the squirmer’s surface consists of radial and tangential surface velocity components:

(7) $$u^{s}(r)=\sum_{n=0}^{\infty }A_{n}\cos(n\theta )\hat{r}+\sum_{n=1}^{\infty }B_{n}\sin(n\theta )\hat{\theta },$$

where $\hat{r}$ and $\hat{\theta }$ are the radial and tangential unit vectors at one point on the squirmer surface. For a more visual illustration, the unit vectors are shown in Figure 1.

**Figure 2:**
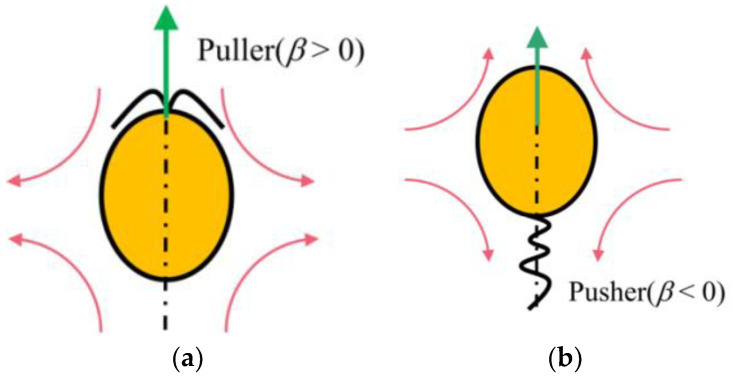
Flow generated by (**a**) a puller and (**b**) a pusher.

For simplicity, the radial component was usually ignored, according to Blake [7]. Under the same assumptions, we adopted the simplified squirmer model represented by the second-order truncated tangential velocity,

(8) $$u^{s}(\theta )=(B_{1}\sin\theta +B_{2}\sin\theta \cos\theta )\hat{\theta }.$$

where *B*_1_ represents the magnitude of the self-propelled force, which is reflected in the final swimming velocity of the squirmer; for example, in the Stokes flow limit, the velocity of a squirmer is *Us* = *B*_1_/2. Note that in all cases, it is necessary to ensure *B*_1_ > 0. Coefficient *B*_2_ it determines the intensity of the vorticity around the squirmer. Here, a swimming type parameter *β* = *B*_2_/*B*_1_ is introduced; the squirmer can be divided into three different types: pullers (*β* > 0), pushers (*β* < 0), and neutrals (*β* = 0), based on the value of *β*. As the name implies, pullers generate thrust from their front (Figure 2a). The pushers push themselves using the cilia at the back of their body, thus generating thrust from the rear (Figure 2b).

### 1.3. Boundary Conditions

In LBM, special treatment of the moving boundary is usually required to ensure no-slip boundary conditions on the surfaces of squirmers. In this work, the improved bounce-back scheme proposed by Lallemand et al. [33] was used and, after improvement, this interpolation-based bounce-back scheme is stable, robust, and easy to implement in simulations.

**Figure 3:**
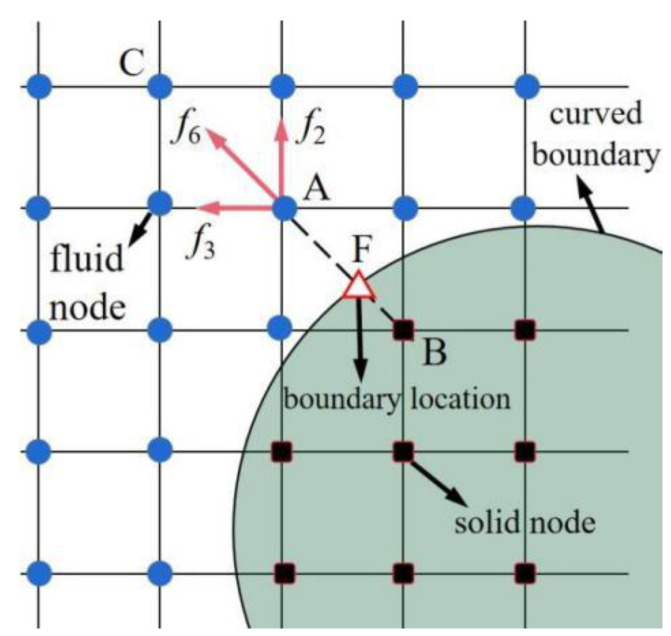
Schematic of the bounce-back scheme in the LBM.

The improved bounce-back scheme is shown in Figure 3, where solid squares indicate solid nodes inside the curved boundary, solid circles indicate fluid nodes outside the curved boundary, and hollow triangles indicate boundary nodes where the bounce-back occurs. Lallemand et al. [33] proposed that all fluid and solid nodes may change after the collision when the distribution function from the solid nodes to the fluid nodes needs to be recalculated based on the exact location of the boundary (i.e., boundary nodes). For example, the distribution function *f*_6_ of the fluid node A originally needs to be calculated by the solid node B. However, node B is inside the curved boundary, so the distribution function f6 cannot be determined. A simple bounce-back scheme can be chosen to satisfy the no-slip boundary condition, such as making *f*_6_ = *f*_8_. However, this method assumes that the boundary node F is located precisely at the midpoint of the line between two lattice nodes, which is not universal. Therefore, an additional calculation of the distribution function *f*_6_ is required. Lallemand et al. [33] introduced the parameter *q*, *q* = |AF|/|AB|, to obtain the location of the boundary nodes more accurately. Then, *f*_6_ can be recalculated based on the near nodes using the interpolation method.

(9) $$f_{6}(A)=\{\begin{array}{l} q(1+2q)f_{8}(B)+(1-4q^{2})f_{8}(A)-q(1-2q)f_{8}(C)-2w_{8}\rho \frac{e_{8}\cdot u_{F}}{c_{s}{}^{2}}q<\frac{1}{2}\\ \frac{1}{q(2q+1)}f_{8}(B)+\frac{2q-1}{q}f_{6}(C)-\frac{2q-1}{2q+1}f_{6}(D)-\frac{2w_{8}}{q(2q+1)}\frac{e_{8}\cdot u_{F}}{c_{s}{}^{2}}q\geq \frac{1}{2} \end{array}.\;$$

where ***u***_F_ is the moving velocity of the surface at the boundary node F, as shown in Figure 3.

The self-propelled motion of the squirrel can be achieved by updating the velocity of the boundary nodes by adding the slip velocity before interpolation, as shown in the following equation,

(10) $$u_{F}=u_{b}+u^{s}=U+\Omega \times R+u^{{}_{s}}.\;$$

where ***U*** and ***Ω*** represent the translational and rotational speed of the squirmer and ***R*** is the position vector starting from the squirmer mass center to boundary node F.

During the calculation, all fluid-solid boundary links will be scanned at each time step, and the force and torque applied to the squirmer can be calculated by the momentum exchange method [34]. In addition, the increased force and torque when the squirmers swim through the fluid and cover the fluid nodes are also addressed [35]. In summary, the motion of the squirmer is defined by solving Newton’s equations using the net force and torque.

(11) $$m\frac{d^{2}x}{dt^{2}}=F$$

(12) $$\frac{d(J\cdot \Omega )}{dt}=T$$

where ***x*** is the center of the squirmer’s mess, ***Ω*** and ***J*** represent the angular velocity and moment of inertia of squirmers, respectively. ***F*** and ***T*** represent the force and torque, respectively, when *ρ_p_* (the density of squirmer) = *ρ* (the density of fluid), the squirmer was neutrally suspended.

### 1.4. Repulsive Force Model

**Figure 4:**
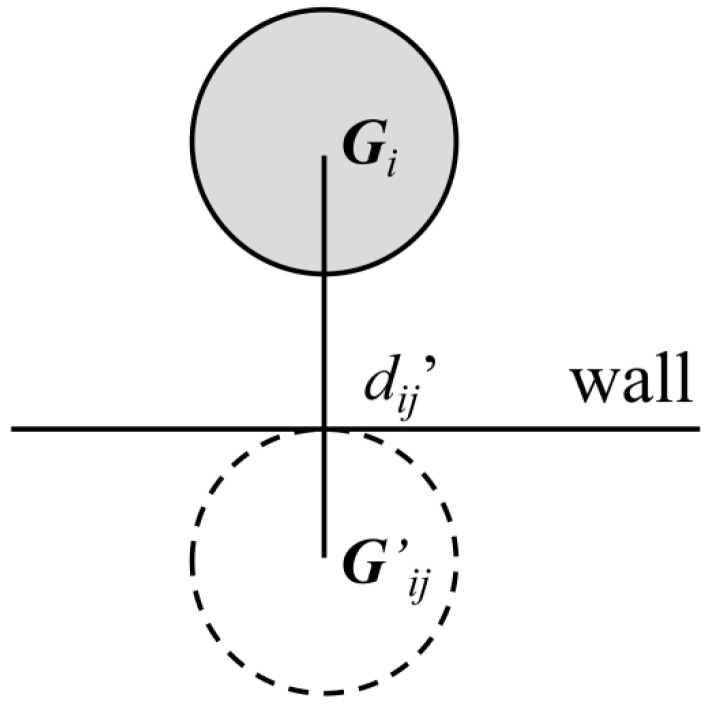
Schematic of the virtual particle.

The distance between squirmers or between the squirmer and wall surface in the simulation may be minimal. In order to prevent particles from penetrating each other or penetrating walls, the collision model proposed by Glowinski et al. [36] was used. *B_i_* and *B_j_* are assumed to be two circular squirmers with radii and centers of mass of *R_i_*, *R_j_*, and ***G****_i_*, ***G****_j_*, respectively. Then, the repulsive force, *F_ij_^p^*, between particles *B_i_* and *B_j_* must satisfy the following equation:

(13) $$F_{ij}^{p}=\left\{ \begin{array}{l} 0\\ \frac{1}{\varepsilon_{p}}{\left(\frac{R_{i}+R_{j}+h_{c}-d_{ij}}{h_{c}}\right)}^{2}\frac{G_{i}G_{j}}{d_{ij}} \end{array}\right.\begin{array}{cc} & \mathrm{if}\\ & \mathrm{if} \end{array}\begin{array}{c} d_{ij}>R_{i}+R_{j}+h_{c}\\ d_{ij}\leq R_{i}+R_{j}+h_{c} \end{array},$$

where *d_ij_* = |***G****_i_**G**_j_*|, *h_c_* is the range of the repulsive force (i.e., the repulsive force occurs at less than *h_c_*), and *ε_p_* is a “small” positive number and is set to 10^−3^. A similar approach is used to deal with the particle-wall interaction by introducing a virtual particle (***G***’*_ij_*), as shown in Figure 4, where *d_ij_’* = |***G****_i_**G***’*_ij_*|. *ε_w_* is similar to *ε_p_*, which is used for the squirmer-wall collision, and the squirmer-wall repulsive force *F_ij_^w^* is given by,

(14) $$F_{ij}^{w}=\left\{ \begin{array}{l} 0\\ \frac{1}{\varepsilon_{w}}{\left(\frac{R_{i}+R_{j}+h_{c}-{d^{\prime }}_{ij}}{h_{c}}\right)}^{2}\frac{G_{i}G_{j}{}^{\prime }}{d_{ij}{}^{\prime }} \end{array}\right.\begin{array}{cc} & \mathrm{if}\\ & \mathrm{if} \end{array}\begin{array}{c} d_{ij}>R_{i}+R_{j}+h_{c}\\ d_{ij}\leq R_{i}+R_{j}+h_{c} \end{array},$$

## 2. Flow and Parameters

**Figure 5:**
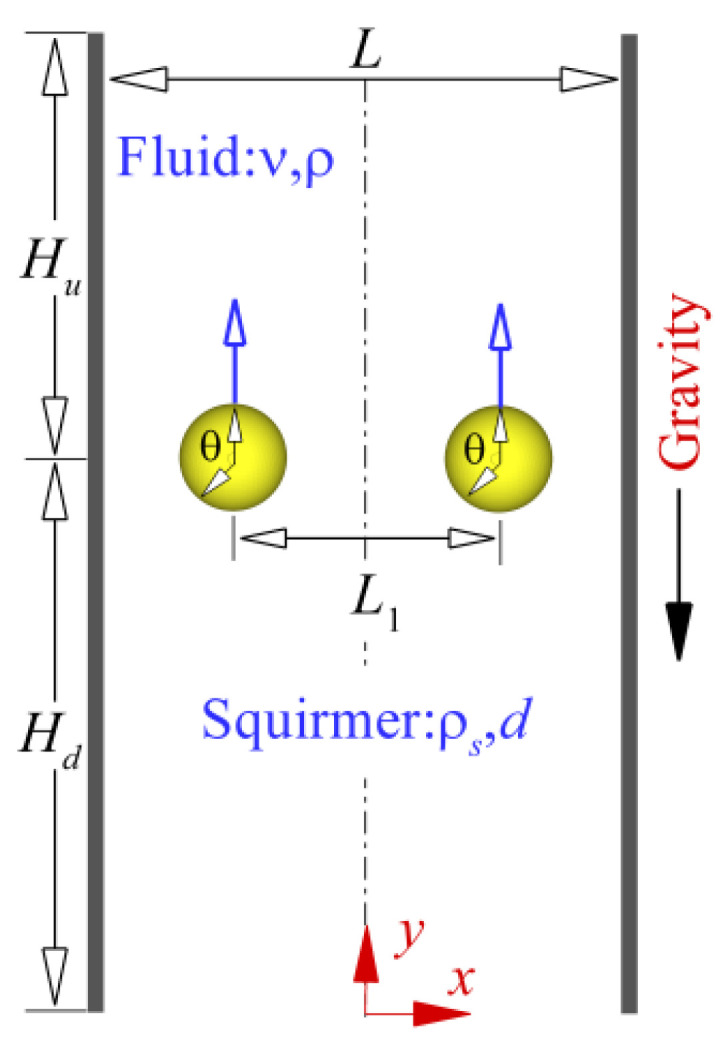
Schematic of two squirmers swimming in a narrow channel.

The swimming pattern of two interacting squirmers under gravity in a narrow vertical channel (Tow-dimensional), as shown in Figure 5, is simulated numerically. Two squirmers with the same diameter *d* and density *ρ_s_* swim in the channel filled with a fluid with density *ρ* and kinematic *ν*. In the simulation, unless otherwise specified, the parameters are fixed as follows: *d* = 24, *L*= 5*d*, *H* =40*d*, initial distance between particles *L*_1_ = 2*d*, *H_u_* = 15*d*, and *H_d_* = 25*d*. The squirmers swim upward (in the opposite direction of gravity) because of *ρ_s_* < *ρ*. The parameters above are all based on lattice units common in the LBM. This study used a moving computational domain to simulate an infinite channel. The Reynolds number is defined as:

(15) $$Re_{g}=\frac{U_{g}d}{\nu }\;,\;$$

(16) $$U_{g}=\frac{\left|\rho_{s}/\rho -1\right|gr^{2}}{4\pi K\nu },$$

where *g* is the gravitational acceleration, *r* is the squirmer radius, *ν* is the kinematic viscosity of the fluid, and *K* is a constant related to the effect of the channel width and is related to the ratio of the channel width to squirmer diameter (W^*^ = *W*/*d*):

(17) $$K=\frac{1}{InW^{*}-0.9157+1.7244/{\left(W^{*}\right)}^{2}-1.7302/{\left(W^{*}\right)}^{4}+2.4056/{\left(W^{*}\right)}^{6}-4.5913/{\left(W^{*}\right)}^{8}}.\;$$

**Figure 6:**
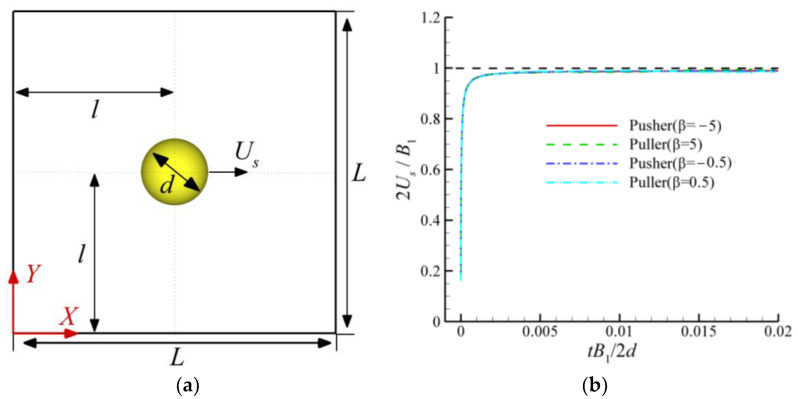
Schematic of the validation of LBM: (**a**) schematic of a squirmer swimming in a square area; (**b**) evolution of the squirmer swimming velocity with time.

As mentioned above, a squirmer’s final steady swimming velocity in a Stokes flow is *B*_1_/2, i.e., *U_s_* = *B*_1_/2. Therefore, the swimming Reynolds number is defined as *Re_s_* = *U_s_d*_1_/*ν*. Additionally, parameter *α* = *U_s_*/*U_g_* indicates the intensity of the squirmer settlement, which describes the competition between the self-propelled force and gravity. The gravity-driven motion of squirmers is dominated by self-propulsion for *α* ≥ 1, called the cruising regime. The motion of the squirmers is dominated by gravity for *α* ≤ 1, referred to as the strong-gravity regime [10]. Parameter *β* = *B*_2_/*B*_1_ is used to indicate the swimming type of the squirmers. Therefore, to observe different dynamic states at low *Re*, *Re*_g_ = 0.1, *α* = [0.1, 1.1], and *β* = [−5, 5] are selected as shown in Figure 6a.

## 3. Validation

To check the accuracy of the LBM, we performed simulations for different *β* at *Re_g_* = 0.1. As shown in Figure 6a, a squirmer with a diameter *d* = 40 moves to the right in a square area of size *L* × *L* = 800 × 800. The densities of the squirmer and fluid are *ρ_f_* = *ρ_s_* = 1, the initial position of the squirmer is in the center of the region at *l* × *l* = 400 × 400, and *Re_s_* = 0.0005. As shown in Figure 6b, the squirmer starts swimming from rest, and all velocities eventually reach the theoretical value of *B*_1_/2, represented by the black dashed line. The numerical simulation results are in good agreement with the theoretical solutions.

**Figure 7:**
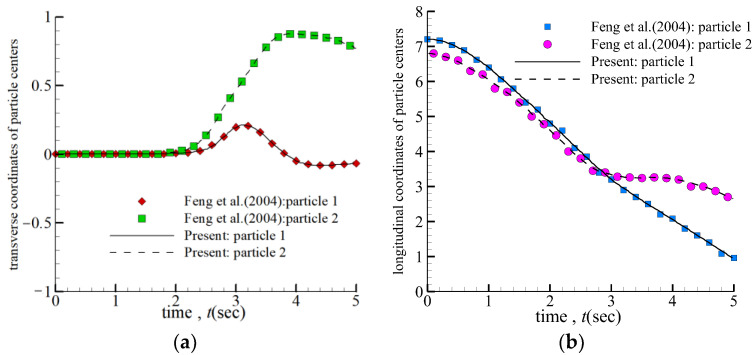
The position coordinates of the centers of the two particles: (**a**) Transverse coordinates; (**b**) longitudinal coordinates, ■: Feng et al. (2004) [37]; ●: Feng et al. (2004) [37].

In addition, to further verify the accuracy of our method in dealing with the interaction between particles. The classical Drifting, Kissing, and Tumbling (DKT) motion of two circular particles were reproduced and compared with the results of Feng et al. [37]. The physical parameters selected are the same as those of Feng et al. [37]. The vertical channel is 20*d* wide (*X*-direction) and 80*d* high (*Y*-direction). The channel is filled with fluid, and the density and kinematic of the fluid are *ρ* = 1 and *ν* = 0.001, respectively. The density and radius of particles are *ρ* = 1.01 and *d* = 50, respectively. The initial velocities of the particles and fluid are zero. At the beginning of the release, the first particle is located 0.01*d* from the center of the channel at a height of 72*d* from the bottom, and the second particle is at the center of the channel at 68*d*. Due to the gravity drive, these two particles start to settle in the *Y*-direction. The repulsive force is added when the gap between particles is less than or equal to 2*d* + ξ and when the gap between the particle and wall is less than 2*d* + ξ. ξ is the range in which repulsion occurs; here, ξ = 1.5Δ*x*. Figure 7a,b shows each particle center’s transverse and longitudinal coordinates, respectively. The comparison shows that our results are in great agreement with the simulation results obtained by previous authors, which proves the correctness of our method.

## 4. Results and Discussion

### 4.1. Swimming Mode

**Figure 8:**
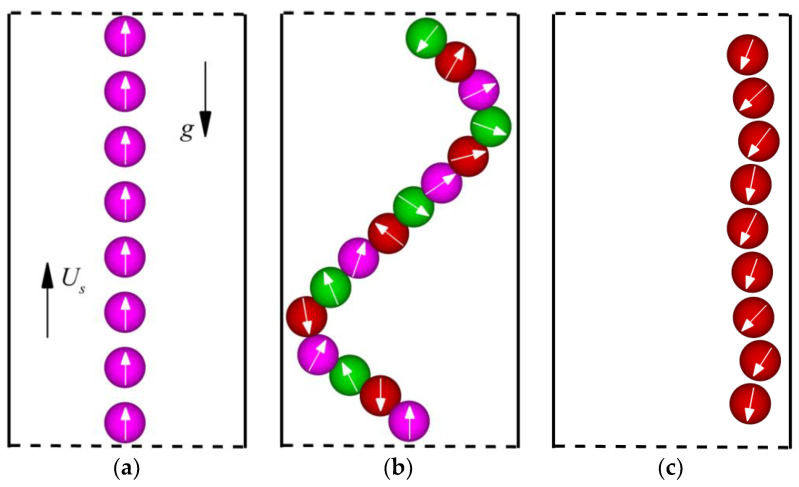

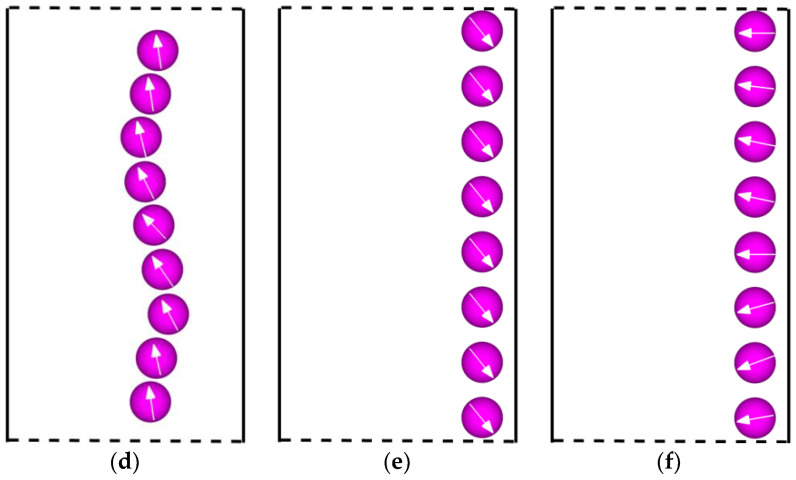
Typical swimming patterns for individual squirmers at different *α* and *β* purple: pullers, red: pushers, green: neutrals). (**a**) Pattern 1: steady upward rising (SUR); (**b**) Pattern 2: oscillation across the channel; (**c**) Pattern 3: oscillation near the wall; (**d**) Pattern 4: vertical motion along the center line with small-amplitude oscillation; (**e**) Pattern 5: vertical motion with invariant orientation near the wall; (**f**) vertical motion with variable orientation near the wall.

When squirmers swim in the channel under different *α* and *β*, their movement modes are diverse, and the collective movement modes depend on the swimming patterns of individual squirmers. Figure 8 shows the typical swimming patterns of individual squirmers. There are five patterns as shown in Figure 8a–e: steady upward rising (SUR) (Pattern 1), oscillation across the channel (Pattern 2), oscillation near the wall (Pattern 3), vertical motion along the center line with small-amplitude oscillation (Pattern 4), and vertical motion with invariant and variable orientation near the wall (Pattern 5). As *α* increases, the swimming velocity of the squirmer dominates, causing the squirmers in patterns 3 and 5 to swim in opposite directions.

**Figure 9:**
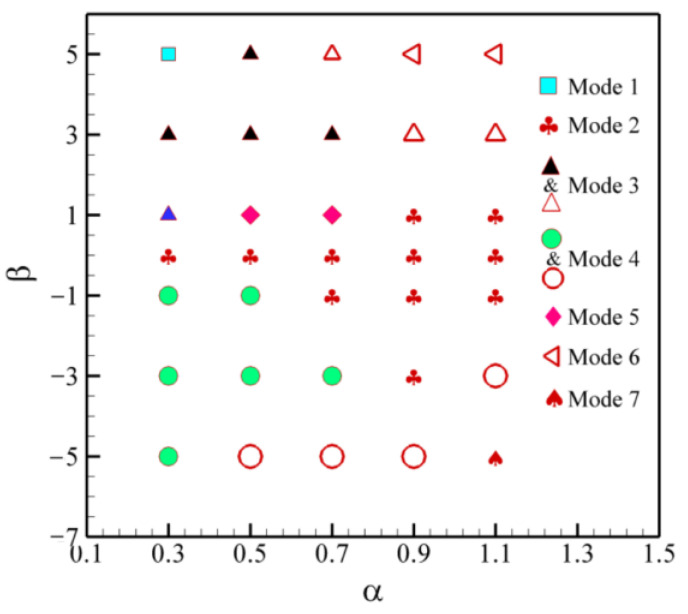
Distributions of swimming modes with different *β* and *α*. Mode 1: composed of Pattern 1; Mode 2: composed of Pattern 2; Mode 3: composed of Patterns 1 and 5; Mode 4: composed of Patterns 2 and 3; Mode 5: composed of Pattern 4; Mode 6: composed of Pattern 5; Mode 7: composed of Pattern 3. The filled and unfilled shapes in Mode 3 and Mode 4 represent the same swimming patterns with different directions.

**Figure 10:**
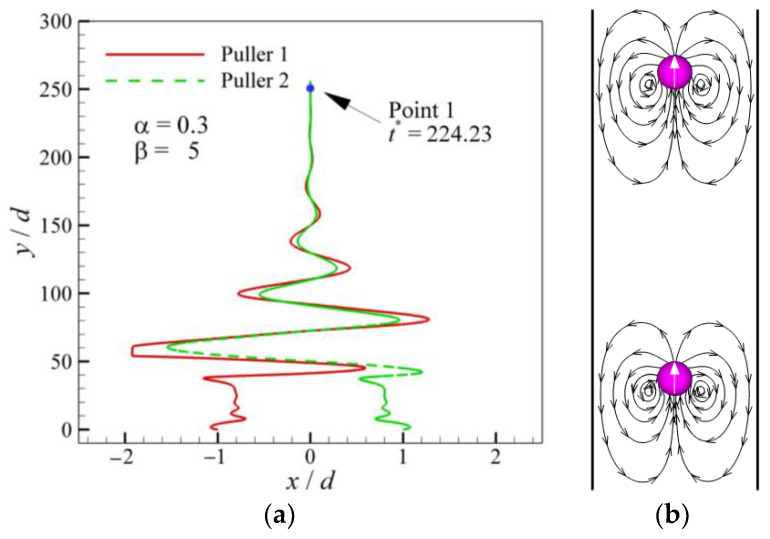
Trajectory and streamlines (solid black lines with arrows) of a pair of pullers (α = 0.3 and *β* = 5): (**a**) trajectory; (**b**) streamlines at t* = 224.23.

Figure 9 shows the swimming modes of a pair of squirmers with different *α* (0.3≤ *α* ≤ 1.1) and *β*(−5 ≤ *β*≤ 5). It is observed that Mode 3, composed of patterns 1 and 5, is located in the region of *β* > 0. As the value of *α* and *β* increases, pattern 5 in Mode 3 changes from vertical up to vertical down, and the triangles change from black-filled to unfilled. The effect of changes in *α* and *β* on Mode 4 is the same as that of Mode 3, but Mode 4 is located in the region of *β* < 0. The swimming mode of all neutral squirmers is Mode 2, which is located in the regions with low *β* (|*β*| ≤ 3) but high *α*(|*α*|≥ 0.7). Mode 1, composed of pattern 1, is located at *α* = 0.3 and *β* = 5, and the trajectory is shown in Figure 8a. It can be seen that the pullers initially repel each other and then attract. After a period of interaction, the final vertical motion occurs in the center of the channel. The streamlines in Figure 10b show that the pullers have reached a delicate balance.

**Figure 11:**
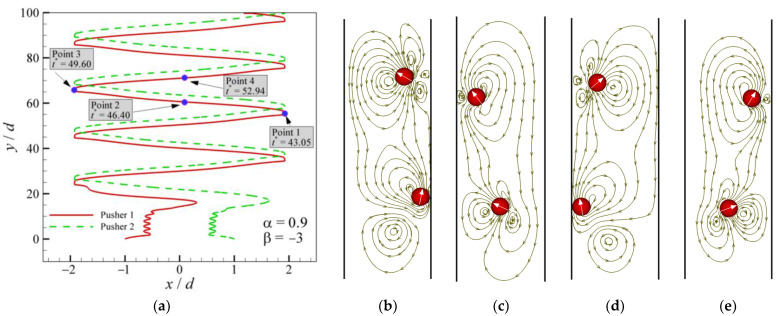
Trajectories and streamlines of a pair of pushers (*α* = 0.9 and *β* = −3): (**a**) trajectories; (**b**) streamlines for Point 1 at t* = 43.05; (**c**) streamlines for Point 2 at t* = 46.40; (**d**) streamlines for Point 3 at t* = 49.60; (**e**) streamlines for Point 4 at t* = 52.94.

**Figure 12:**
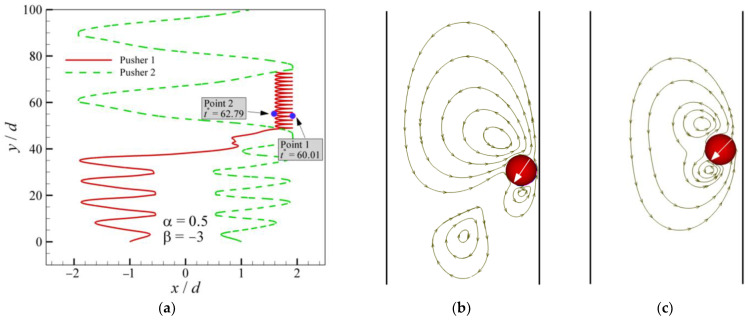
Trajectories and streamlines of a pair of pushers (*α* = 0.5 and *β* = −3). (**a**) trajectories; (**b**) streamlines for Point 1 at t* = 60.01; (**c**) streamlines for Point 2 at t* = 62.79.

As previously described, the patterns of OAC are observed for all types of squirmers. Mode 2 at *α* = 0.9 and *β* = −3 is shown in Figure 11. The trajectory (Figure 11a) shows that two pushers attract each other until they lean together, after which there is a period of interaction. Eventually, they swim in a pattern from the leftmost end of the channel to the rightmost, i.e., OAC. As shown in Figure 11b–e, when the squirmers swim toward the wall, the swimming direction of the squirmers always points to the wall. When the squirmers come into contact with the wall, the swimming direction turns to the other side wall until making contact with the wall. The main difference between squirmers and conventional particles is the self-propelled direction, which can also be called the head. The trajectories and streamlines of Mode 4 are shown in Figure 12. One pusher swims near the wall while the other swims oscillate across the channel. Therefore, they are subjected to different swimming resistance, eventually causing the latter to leave the former far behind. When the former squirmer is closest to and farthest from the wall, the streamlines are presented in Figure 12b,c, where the wall pulls the squirmer back when the pusher tries to swim away from the wall.

### 4.2. Reasons for Different Modes

**Figure 13:**
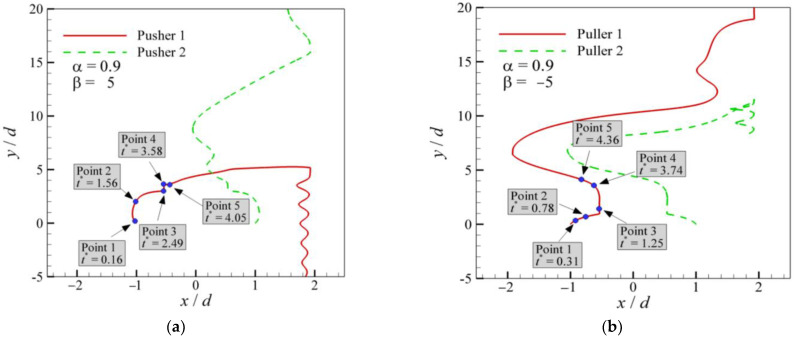
Trajectories of pairs of squirmers: (**a**) pullers at *α* = 0.9 and *β* = 5; (**b**) pushers at *α* = 0.9 and β= −5.

The trajectories of the two squirmers at *α* = 0.9 and different *β* values are presented in Figure 13. The squirmers exhibit an utterly different swimming behavior in the initial swimming phase because of the different *β*. For pullers (*β* = 5), as shown in Figure 13a, two pullers appear to repel at the beginning of the rising movement and then approach each other until fitting together. When the pullers fit together, the dynamic equilibrium between the two pullers is broken as the pullers continue to swim, which causes a large deflection in the horizontal position and results in various subsequent swimming modes. For the pushers (*β* = −5), as shown in Figure 13b, two pushers attract and move closer to each other, and then there is a period of equilibrium. Its duration is longer for pushers than that for pullers. The equilibrium is broken, resulting in a large deflection in the horizontal position, leading to different subsequent swimming modes.

**Figure 14:**
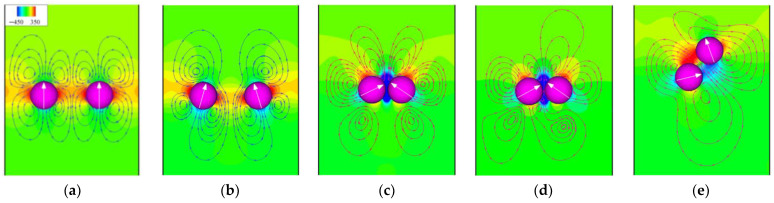
Instantaneous distribution of pressure and streamlines for pullers at *α* = 0.9 and *β* = 5. (**a**) t* = 0.16; (**b**) t* = 1.56; (**c**) t* = 2.49; (**d**) t* = 3.58; (**e**) t* = 4.05.

**Figure 15:**
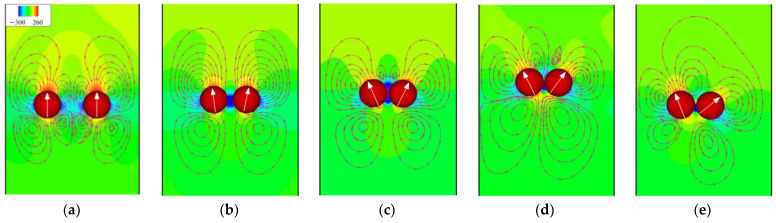
Instantaneous distribution of pressure and streamlines for pushers at *α* = 0.9 and *β* = −5. (**a**) t* = 0.31; (**b**) t* = 0.78; (**c**) t* = 1.25; (**d**) t* = 11.74; (**e**) t* = 12.36.

**Figure 16:**
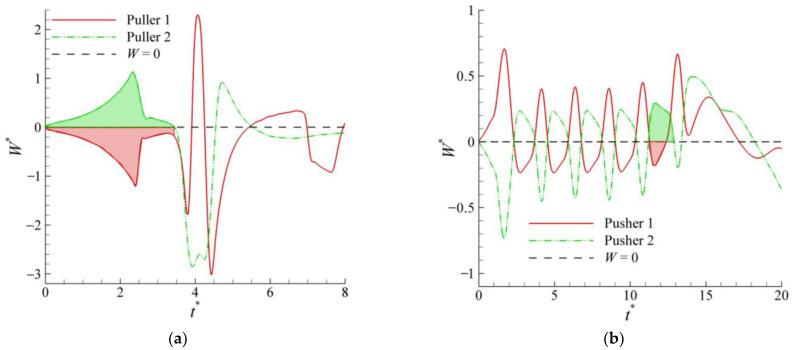
Evolution of the squirmer angular velocities (**a**) pushers at *α* = 0.9 and *β*= −5; (**b**) pullers at *α* = 0.9 and *β* = 5 (asterisk represents the normalized quantity).

To better understand the differences in the initial motions of the two pullers and pushers and the reasons for the loss of equilibrium of the two squirmers, the streamlines and pressure distribution are shown in Figure 14 and Figure 15, respectively. For pullers, the two sides of the pullers are high-pressure regions because a puller pulls the fluid to the sides of its body. In contrast, the upper and lower ends are low-pressure regions, which also causes the two pullers to repel each other initially (Figure 14a). However, at this time, the angular velocity of puller 1 on the left is negative (counterclockwise direction is positive), as shown in Figure 16a, thus making puller 1 rotate in a clockwise direction. In contrast, puller 2 on the right rotates in a counterclockwise direction, and thus the pullers’ swimming directions gradually point toward each other (Figure 14b). At this time, the high-pressure region between the pullers becomes a low-pressure region, while the repulsive force between them also becomes an attractive force. When the two pullers are close together, a low-pressure region is formed between them (Figure 14c), and equilibrium is maintained. However, as the pullers continued to swim, puller 2 suddenly acquired a greater negative angular velocity (Figure 16a), thereby breaking the equilibrium.

The heads and tails of the pushers are located in high-pressure regions because a pusher pushes the fluid toward the head and tail of its body, whereas the sides of their bodies are low-pressure regions. The opposite is true for pullers. The low-pressure region causes two pushers to be attracted to each other initially. Unlike the pullers, the angular velocity of pusher 1 on the left is favorable at this time (Figure 15b), i.e., it rotates counterclockwise. In contrast, pusher 2 on the right rotates clockwise. Then, two pushers swim backward, but the low-pressure region between them continues to pull them toward each other, with an equilibrium similar to that of the pullers. This back-to-back dynamic equilibrium is more stable than the head-to-head equilibrium, resulting in a more prolonged equilibrium for the pushers, as shown in Figure 16. However, as the pushers continue to swim, pusher 2 will gain a more positive angular velocity because there is a slight fluid disturbance, thus breaking the equilibrium.

**Figure 17:**
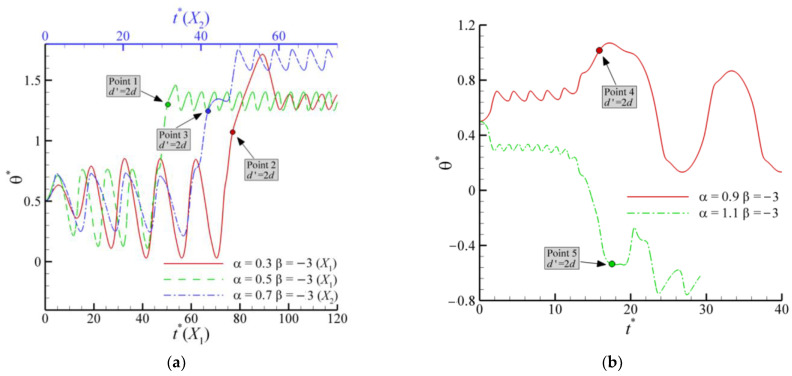
Evolution of orientation angles of pushers with β = −3 at different α: (**a**) α = 0.3–0.7; (**b**) α = 0.9–1.1. The breakdown of equilibrium will result in different distributions of two pushers in the vertical position. For simplicity, the pusher located at the latter is chosen as the object of study, and the initial θ* is 0.5.

**Figure 18:**
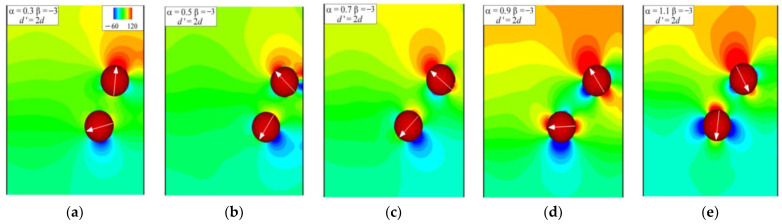
Instantaneous pressure distributions and squirmer orientation for β = −3 at different α at distance d’ = 2d where d’ is the distance between the centers of the two squirmers, (**a**) α= 0.3; (**b**) α = 0.5; (**c**) α = 0.7; (**d**) α = 0.9; (**e**) α = 1.1.

When the dynamic equilibrium between the two squirmers is broken, their interaction continues until they separate from each other and swim separately. Moreover, the squirmer orientation and pressure distribution after the squirmers’ interaction determine their subsequent swimming patterns. Here, the parameter *d*’ = 2*d* is defined, i.e., when the distance between the centers of the two squirmers is larger than *d*’, the interaction between the two squirmers ends. In Figure 17, the points for *d*’ = 2*d* are marked. For *α* = 0.3, 0.5, and 0.7 (Figure 17a), the swimming pattern of the pushers is pattern 3 (Figure 8c), and the swimming angles all change in a homeopathic manner before contacting the wall, indicating the feasibility of *d*’. The instantaneous pressure distributions and squirmer orientation for *α* = 0.3, 0.5, and 0.7 are shown in Figure 18a–c, where the distribution and graph are similar. It can be seen that there is a low-pressure region in the lower right of the swimming direction of the pushers, which is the cause of the pusher’s counterclockwise rotation. This low-pressure region is attracted to the wall, thus pulling the pushers towards the wall and eventually forming the swimming pattern 3 (Figure 8c). When *d*’ = 2*d* for *α* = 0.9, the swimming direction of the pusher is nearly horizontal, the head and tail of the pusher are located in the high-pressure regions, and the low-pressure region is located below the body (Figure 18d), which allows the pusher to remain stable until it comes in contact with the wall. Eventually, the pusher interacts with the wall and forms the swimming pattern 2. When *α* increases to 1.1, the pressure distribution around the pusher is similar to that for *α*= 0.3, 0.5, and 0.7, as shown in Figure 18e. However, at this point, the pusher has a greater swimming intensity, thus generating the same swimming pattern but in the opposite direction.

**Figure 19:**
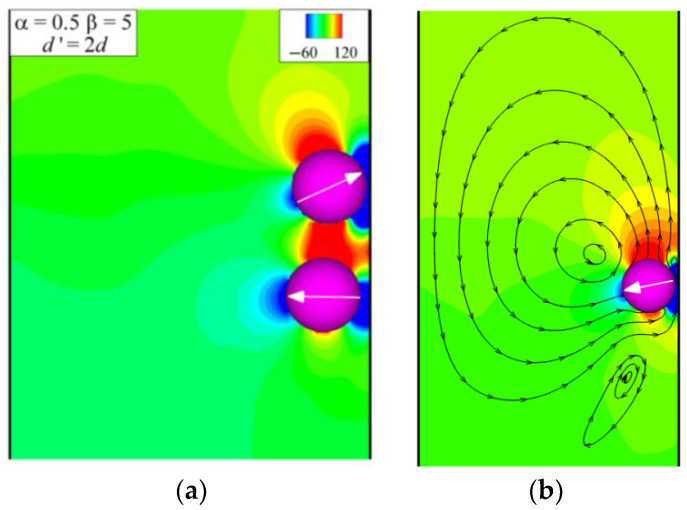
Instantaneous pressure distribution and squirmer orientation for α = 0.5 and β = 5: (**a**) point at which d’ = 2d; (**b**) pressure distribution and streamlines for a single puller.

For the pullers, the situation is different. The low-pressure region at the head of the below puller is attracted to the low-pressure region of the tail of the above puller, as shown in Figure 14e when the dynamic equilibrium is broken, which is opposite to the situation for the pushers shown in Figure 15e. Therefore, for pullers, there will be a more extended interaction period before the two pullers are entirely separated. Taking *α*= 0.5 and *β*= 5 as an example, when the two pullers swim near the wall at *d*’ = 2*d*, the low-pressure region at the head of the above puller and the tail of the below puller are both attracted by the wall, while there is a high-pressure region between the two pullers as shown in Figure 19a. These different forms of interaction with the wall cause their subsequent different swimming patterns, as shown in Figure 19b, where the puller swims vertically upward near the wall, as shown in Figure 8f.

## 5. Conclusions

The interaction between a pair of squirmers subject to gravity and the swimming mode of squirmers in a narrow vertical channel (2-D) are investigated in the range of 0.1 ≤ *α* ≤ 1.1 and −5 ≤ *β* ≤ 5. The main conclusions are summarized below.

The swimming modes of the two interacting squirmers are the combination of the two squirmers’ independent swimming patterns. The main swimming patterns are different for different types of squirmers. Neutral squirmers exhibit the pattern of OAC. The main swimming pattern for pullers is a combination of vertical motion along the center and near the wall. The main swimming pattern for pushers is a combination of OAC and ONW.

A pair of pullers swimming upward side-by-side will repel each other because of the high-pressure region between them. The pressure distribution changes with the deflection of the squirmer orientations, and the high-pressure region becomes a low-pressure one, resulting in subsequent attraction and close contact between the squirmers. The initial low-pressure region between the pushers causes them to be attracted and come into contact.

Lastly, the swimming modes in the range between 0.1 ≤ *α* ≤ 1.1 and −5 ≤ *β* ≤ 5 were summarized, and is was found that the different swimming modes are related to the rotation angles and the swimming velocities (after separation) of the two squirmers. Comparing the pushers at *α* =0.3 and *α* = 0.5, when *β* = −3, it can be seen that *α* = 0.3 has a smaller rotation angle (in the horizontal direction) and thus has a more significant horizontal velocity; while *α* = 0.5 is the opposite, leading to a transition between oscillation across channel and oscillation near the wall. A similar outcome was observed at *α* =0.5 and *α* = 0.7.

## Data Availability

Not applicable.
